# Nanostructured Lipid Carrier Co-Loaded with Docetaxel and Magnetic Nanoparticles: Physicochemical Characterization and In Vitro Evaluation

**DOI:** 10.3390/pharmaceutics15051319

**Published:** 2023-04-22

**Authors:** Auni Hamimi Idris, Che Azurahanim Che Abdullah, Nor Azah Yusof, Azren Aida Asmawi, Mohd Basyaruddin Abdul Rahman

**Affiliations:** 1 Faculty of Chemical and Process Engineering Technology, Universiti Malaysia Pahang, Lebuh Persiaran Tun Khalil Yaakob, Kuantan 26300, Pahang, Malaysia; 2 Integrated Chemical BioPhysics Research, Faculty of Science, Universiti Putra Malaysia, Serdang 43400, Selangor, Malaysia; azurahanim@upm.edu.my (C.A.C.A.);; 3 UPM-MAKNA Cancer Research Laboratory, Institute of Bioscience, Universiti Putra Malaysia, Serdang 43400, Selangor, Malaysia; 4 Institute of Nanoscience and Nanotechnology, Universiti Putra Malaysia, Serdang 43400, Selangor, Malaysia; azahy@upm.edu.my

**Keywords:** docetaxel, lipid-based carrier, nanostructured lipid carrier, theranostic

## Abstract

Lung cancer is currently the most prevalent cause of cancer mortality due to late diagnosis and lack of curative therapies. Docetaxel (Dtx) is clinically proven as effective, but poor aqueous solubility and non-selective cytotoxicity limit its therapeutic efficacy. In this work, a nanostructured lipid carrier (NLC) loaded with iron oxide nanoparticles (IONP) and Dtx (Dtx-MNLC) was developed as a potential theranostic agent for lung cancer treatment. The amount of IONP and Dtx loaded into the Dtx-MNLC was quantified using Inductively Coupled Plasma Optical Emission Spectroscopy and high-performance liquid chromatography. Dtx-MNLC was then subjected to an assessment of physicochemical characteristics, in vitro drug release, and cytotoxicity. Dtx loading percentage was determined at 3.98% *w*/*w*, and 0.36 mg/mL IONP was loaded into the Dtx-MNLC. The formulation showed a biphasic drug release in a simulated cancer cell microenvironment, where 40% of Dtx was released for the first 6 h, and 80% cumulative release was achieved after 48 h. Dtx-MNLC exhibited higher cytotoxicity to A549 cells than MRC5 in a dose-dependent manner. Furthermore, the toxicity of Dtx-MNLC to MRC5 was lower than the commercial formulation. In conclusion, Dtx-MNLC shows the efficacy to inhibit lung cancer cell growth, yet it reduced toxicity on healthy lung cells and is potentially capable as a theranostic agent for lung cancer treatment.

## 1. Introduction

Until 2020, global lung cancer prevalence was estimated to increase to 2.2 million cases compared to 2.1 million cases in 2018 [1]. The exact cause of lung cancer is unknown, but a high-risk lifestyle such as cigarette smoking has been identified as a major risk factor. Symptoms of lung cancer are similar to other respiratory and lung diseases, which complicates a correct diagnosis; thus, it is often confirmed when the patients have reached stages III and IV. According to Sung et al. [1], it was estimated that most lung cancer patients die within one year of diagnosis, and only 18% survive within five years due to poor prognosis and less effective treatment. Standard therapy for lung cancer patients includes surgery, chemotherapy, and radiotherapy. Surgery and radiotherapy are often combined with chemotherapy to avoid recurrent tumor progression for more effective treatment. Anticancer agents such as doxorubicin, cisplatin, and docetaxel (Dtx) have been shown to inhibit solid tumor progression in the lungs, breasts, and prostates, among others [2]. Nevertheless, clinical applications of these drugs are limited due to low aqueous solubility, serious side effects, and non-specific distribution of the body. For example, the commercial formulation of Dtx (Taxotere^®^) contains a high amount of surfactant (Tween 80) to increase its aqueous solubility. Although this formulation is proven effective in cancer therapy, it has caused dose-limiting toxicity and hypersensitivity reactions in patients, as both cancer cells and healthy cells are exposed to the toxicity effects, leading to severe adverse effects such as neurotoxicity and neutropenia [3].

Nanoparticles formulated from solid lipids, e.g., SLN, present high stability in vivo and often demonstrate controlled release kinetics [4]. SLN offers many advantages compared to other nanoparticle systems, as a wide range of biocompatible materials is available to suit the application. SLN is solid at room and body temperatures due to the solid lipid used in the preparation process. It is generally composed of 0.1–30% (*w*/*w*) of lipid and 0.5–5% (*w*/*w*) of surfactant to stabilize lipid dispersion in aqueous media [5]. However, polymorphic change in the solid lipid structure during lipid recrystallization was shown to affect drug loading and potentially cause drug removal from the lipid matrix during storage, consequently limiting their potential as DDS [6]. This instability issue has led to an improvement in the SLN by blending solid lipids with oil to create a nanostructured lipid carrier. In 2002, Müller et al. [7] started incorporating liquid lipid in the solid lipid to form NLC, which resulted in higher loading capacity and better stability than SLN. NLC’s solid state in room and body temperature remains unchanged, but studies showed that better drug loading capacity, greater storage stability, and release behavior modulation of the therapeutic payload were achieved due to the less orderly lipid crystal structure compared to SLN [8,9]. The types of lipid and the ratio of solid and liquid lipid selected to produce NLC were observed to significantly impact their polymorphism, possible existence of supercooled melts, and the presence of other colloidal species. Therefore, careful selection of formulation excipients and investigations on their physicochemical properties are required to develop a suitable formulation designed for lung cancer therapy.

NLC can also be co-loaded with imaging agents for combined therapeutic and diagnostic purposes, termed theranostics. Imaging modalities such as fluorescence imaging and magnetic resonance imaging (MRI) are helpful to visualize biological processes (e.g., apoptosis and cell trafficking), for monitoring responses to therapy and for diagnosing diseases [10,11,12]. The entrapment of imaging agents in its lipid core will enable signal enhancement in the targeted tissue after cell uptake. For instance, in situ visualization of the cellular uptake of the SLN/siRNA complexes was made possible by co-loading the SLN with paclitaxel and quantum dots [13]. Olerile et al. reported that tumor detection and therapeutic activity in the murine model were improved by embedding paclitaxel and quantum dots in their NLC formulation [14]. The incorporation of IONP in NLC formulation enables tumor imaging using MRI, due to its magnetic behavior in the presence of a magnetic field. Several works have demonstrated in vivo imaging of hepatic tumors in the mouse model by incorporating IONP as a T_2_-weighted MRI contrast agent in NLC [15,16]. The potential use of SLN formulations containing Prostacyclin and IONP as image-guided therapy in atherosclerosis was demonstrated by the high transverse relaxivity of the IONP-loaded SLN [17]. Therefore, the theranostic application of NLC can be achieved by dual loading of active ingredients and imaging agents such as IONP in the formulation for higher detection sensitivity and monitoring therapy efficacy.

Current chemotherapy effectiveness can only be determined upon treatment completion by physical examinations, X-ray/Computed Tomography (CT) scan, or blood tests. The treatment protocol would be changed later if inadequate chemotherapy response was measured, which consequently could cause the tumor to progress further before an effective treatment regime can be prescribed. Theranostics in oncology offers unique opportunities to provide diagnostic imaging and therapeutic molecules in a single platform. Functionalization of nanocarriers with magnetic nanoparticles such as iron oxide nanoparticles (IONP) will enable visualization of tumors and metastases in various organs such as the liver, spleen, and lymph nodes using Magnetic Resonance Imaging (MRI) [18]. A theranostic approach to continuously monitor the treatment response is beneficial for measuring chemotherapy effectiveness.

The present study focuses on the physicochemical characterization of a nanostructured lipid carrier co-loaded with docetaxel and magnetic nanoparticles with the aim to improve the bioavailability and to reduce the toxicity associated with Dtx formulation in lung cancer treatment. Comparison of the in vitro cytotoxicity of Dtx-MNLC and the commercialized formulation of Dtx was carried out on normal human lung fibroblast cell lines and adenocarcinoma epithelial cell lines.

## 2. Materials and Methods

### 2.1. Materials

Iron(III) oxide(FeO(OH); hydrated, catalyst grade, 30–50 mesh; cat. #371254 oleic acid (technical grade, 90%), 1-octadecene (ODE; technical grade, 90%), and fish oil were purchased from Sigma-Aldrich (Steinheim, Germany). Docetaxel (Dtx; >98% purity) and trifluoroacetic acid (99% extra purity) were provided by Acros Organics (Fair Lawn, NJ, USA). Sodium 3′-[1-(phenylaminocarbonyl)-3,4-tetrazolium]-bis (4-methoxy 6-nitro) benzene sulfonic acid hydrate (XTT sodium salt) was purchased from GoldBio (St. Louis, MO, USA). Acetone and acetonitrile (HPLC grade) were purchased from Fisher Scientific (Loughborough, UK), while methanol (99.9% purity) was purchased from Merck (Darmstadt, Germany). Lipids including Precirol ATO 5 and Caprylic/Capric triglyceride (MCT; Labrafac CC) were provided by Gattefossé (Saint-Priest, France). Surfactants including lecithin Lipoid S75 and Vitamin E TPGS were provided by Lipoid GmbH (Ludwigshafen, Germany) and Sigma-Aldrich (Steinheim, Germany), respectively. Simulated lung fluid was purchased from R&M Chemicals (Selangor, Malaysia). The materials were all analytical grade and used without additional purification unless otherwise stated.

### 2.2. Preparation of Iron Oxide Nanoparticles (IONP)

The oleic acid-coated IONP (OA-IONP) was synthesized according to the modified procedure by Yu et al. [19]. In brief, 0.534 g (6 mmol) FeO(OH), 6.78 g (24 mmol) oleic acid, and 15 g (59 mmol) ODE were mixed in a 100 mL three-neck round bottom flask and magnetically stirred under the flow of nitrogen at 100 °C for 10 min. The mixture was then heated to reflux at 320 °C for 1 h before cooling to room temperature. The resulting brown-black colloid was collected and purified several times with a mixture of hexane/acetone (1:4 *v*/*v*) and centrifuged to remove excess reactants. The OA-IONP was finally dried in an oven at 80 °C for 24 h to obtain a black powder. The OA ligand was then substituted with fish oil as a ligand using a modified ligand exchange procedure [20], for further loading into the magnetic nanostructured lipid carrier (MNLC).

### 2.3. Preparation of Magnetic Nanostructured Lipid Carrier (MNLC)

A certain amount of IONP was dispersed in distilled water and sonicated in a bath sonicator for 15 min to ensure homogeneous dispersion. The IONP–distilled water mixture was subsequently heated to 60 °C and stirred using an overhead stirrer at 400 rpm for 20 min. In a separate container, Dtx, Lipoid S75, and TPGS were weighed and heated to 60 °C while shaken at 300 rpm in the Thermomixer Comfort. Once melted, a mixture of ethanol/acetone (70:30 *v*/*v*) was added into the container. This ethanol/acetone mixture was then rapidly injected into the distilled water mixture using a syringe fitted with a 20G × 1½ “ hypodermic needle. The mixture was stirred continuously at 60 °C to form an emulsion and to evaporate the organic solvent. The emulsion was immediately transferred into a 15 mL centrifuge tube and immersed in an ice bath (4 °C) for one hour to solidify the Dtx-MNLC. Further stabilization of the lipid nanoparticles was carried out at room temperature for 24 h. Dtx-MNLC was purified using a combination of centrifugation and dialysis methods to remove unreacted emulsifier and organic solvent. The formulation was first centrifuged at 4000 rpm for 15 min to precipitate unentrapped IONP. A dialysis bag with 12–14 kDa MWCO was filled with the Dtx-MNLC and the formulation was dialyzed against distilled water for 12 h with one change of dialysate after the first 4 h. The Dtx-MNLC was finally collected into a 50 mL centrifuge tube.

### 2.4. Physicochemical Characterisation

#### 2.4.1. Morphology and Particle Size Analysis

The morphology of Dtx-MNLC was observed on a high-resolution Transmission Electron Microscope (JEOL JEM-2100F) operating at 80 kV. The samples were first diluted with distilled water and negatively stained with uranyl acetate. Samples were then allowed to dry on a carbon-coated copper grid at room temperature. Particle size was determined by calculating the diameter, *D*, from the area of each particle, *A*, measured in ImageJ using Equation (1). Approximately 100 particles were measured to present a good estimation of size distribution.

(1) $$D=\left(\sqrt{\frac{A}{\pi }}\right)\times 2$$

The particle size measured from HRTEM images was compared with the mean particle size measured using Zetasizer (Nano ZS90, Malvern Instruments, Worcestershire, UK). The samples were prepared by diluting 50 μL of Dtx-MNLC in 1 mL of deionized water and injection into the sample cell. The sample was first equilibrated for 20 s upon loading the sample cell into the instrument. The results were averaged over three experiments.

#### 2.4.2. Entrapment Efficiency and Drug Loading

The entrapment efficiency of Dtx was determined from the precipitated unentrapped drug recovered from centrifugation of the formulation at 4000 rpm. The precipitate was dissolved in 3 mL of methanol and mixed using a vortex mixer for 2 min. This mixture was centrifuged at 4000 rpm for 15 min to precipitate the IONP. The supernatant was filtered using a 0.45 μm PTFE syringe filter and the amount of unentrapped Dtx was determined using HPLC-UV.

The amount of Dtx loaded in the formulation to calculate the drug loading percentage was determined according to Naguib et al. [21]. An amount of 0.5 mL of Dtx-MNLC was mixed with 2.5 mL methanol for 30 min at 65 °C using a Thermomixer Comfort to dissolve the lipid. The mixture was then immediately placed at −20 °C for one hour and centrifuged at 6000 rpm (4 °C) for 15 min to precipitate the solid lipid. The supernatant was filtered using a 0.45 μm PTFE syringe filter and the concentration of Dtx was determined using HPLC-UV. The following equation was used to calculate the percentage of entrapment efficiency (EE) and drug loading (DL) in the Dtx-MNLC.

(2) $$\mathrm{Entrapment}\;\mathrm{Efficiency}\;\left(\%\right)=\frac{W_{1}-W_{2}}{W_{1}}\;\times \;100\;$$

(3) $$\mathrm{Drug}\;\mathrm{loading}\;\left(\%\right)=\frac{W_{3}}{W_{L}}\;\times \;100$$

where *W*_1_ is the initial amount of drug added to the Dtx-MNLC, *W*_2_ is the amount of drug in the precipitate, *W*_3_ is the amount of drug in the purified MNLC, and *W_L_* is the amount of lipid in the Dtx-MNLC.

Quantification of Dtx was carried out using an HPLC system (Waters Corporation, Milford, MA, USA) equipped with dual GM150 gradient mixers in the 1525 pump, a Waters 2489 UV Visible detector, a reverse phase C18 analytical column (Eclipse Plus, Agilent Technologies, Santa Clara, CA, USA) with a particle size of 5 μm, a length of 150 mm, and a diameter of 4.6 mm, and Breeze software. The detection of Dtx was carried out at the wavelength of 230 nm and a flow rate of 1.0 mL/minute. A mobile phase containing acetonitrile and distilled water at the ratio of 55:45 (*v*/*v*) was adjusted to pH 3 by adding 0.01% trifluoroacetic acid (TFA).

#### 2.4.3. Iron Content Analysis

The iron content in Dtx-MNLC was determined to estimate the amount of IONP entrapped in the lipid carrier following a method by Costo et al. [22]. The elemental analysis was performed on the inductively coupled plasma-optical emission (ICP-OE) spectrometer using Optima 2000™ (Perkin-Elmer Instruments, Shelton, CT, USA) at a wavelength of 238.204 nm. The samples were prepared as follows: 800 μL Dtx-MNLC was pipetted into a 10 mL glass test tube. An amount of 300 μL of 37% hydrochloric acid (HCl) was added into the same tube and heated to 80 °C for one hour using a Thermomixer Comfort with agitation at 300 rpm to ensure complete dissolution of iron. The solution was then transferred into a 25 mL volumetric flask and was diluted with deionized water to the mark for the analysis. For the iron quantification, a 3-point calibration line was developed between 1 to 10 ppm before sample measurement. Direct dilution was carried out from a stock solution of 2 mg/mL IONP (coated with fish oil) in deionized water to prepare standard samples of 5, 7, and 10 ppm. Samples’ digestion with 37% HCl and dilution in a 25 mL volumetric flask was conducted exactly in the same way as sample preparation for Dtx-MNLC.

#### 2.4.4. Thermal Analysis

The thermal behavior of Dtx-MNLC was studied using DSC to determine their melting point and heat of fusion. Prior to analysis, the sample was weighed and placed in an aluminum pan and crimped to seal the sample. The thermal analysis was performed on a DSC (Perkin-Elmer) over a temperature range of 4–300 °C at a scanning rate of 10 °C/min. During the measurement, nitrogen gas was purged at a pressure of 2 bar flowing at 50 mL/min.

#### 2.4.5. Crystallinity Study of Dtx-MNLC

The crystallinity of the formulation was studied using powder X-ray diffraction (PXRD). The sample was first lyophilized, and the dried sample was packed into a sample holder before measurement. The X-ray diffraction patterns were obtained with a Shimadzu XRD-6000 diffractometer using Cu Kα radiation of λ = 1.54056 Å operated at 30 kV and 30 mA. Data were collected from 4–40° with a scan rate of 4°/min.

#### 2.4.6. Fourier Transform Infrared (FTIR) Spectroscopy

Attenuated Fourier Transform Infrared (ATR-FTIR) was used to verify the entrapment of IONP and Dtx inside the formulation. The sample was loaded on a sample container of a Nicolet 6700 FTIR spectrometer (Thermo Scientific, Waltham, MA, USA) and measured in the scanning range of 400–4000 cm^−1^.

### 2.5. In Vitro Drug Release Study via Dialysis

**Figure 1:**
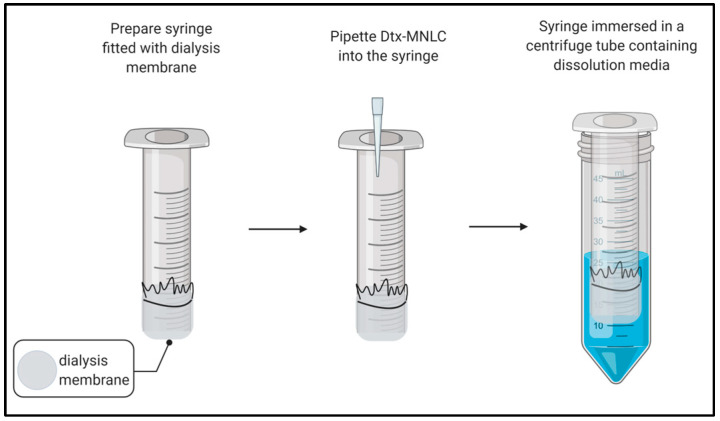
Schematic of drug release studies experimental setup.

In vitro drug release from Dtx-MNLC was performed using a dialysis method in a simulated lung fluid (SLF) at pH 7.4 and 6.0, each containing 0.5% (*w*/*v*) Tween 80 as the dissolution media [23]. Fisherbrand™ regenerated cellulose dialysis tubing with molecular weight cutoff 12–14 kDa (Fisher Scientific) was used as the membrane in the drug release studies. Before the release studies, the dialysis tubing was prepared as follows: the dialysis tubing was first washed thoroughly under running water and immersed for 2 min in hot water (60 °C). The tubing was then soaked overnight in the release media. The membrane was cut and fixed at the bottom of the 3 mL syringe using a rubber band. The experimental setup was adapted from glass basket dialysis as shown in Figure 1 [24]. In this study, 3 mL syringes were used as the ‘basket’. The release of Dtx-MNLC was compared with a Dtx solution from a commercially available formulation (Taxotere^®^). The Dtx solution was prepared by dissolving Dtx in distilled water containing 25% *w*/*v* Tween 80 and 9.75% *v*/*v* ethanol to obtain a concentration of 10 mg/mL Dtx [23]. Both samples were diluted with release media prior to release studies to obtain a final concentration of 0.5 mg/mL Dtx. The experiment was performed using a Thermomixer Comfort at 37 ± 1 °C with agitation at 300 rpm for 48 h. In order to maintain the sink condition, 0.5 mL of the sample aliquot was taken out and replaced with an equivalent volume of fresh dissolution media at predetermined intervals. The drug concentration in the collected samples was then analyzed using the HPLC method as described previously.

### 2.6. In Vitro Cytotoxicity Assessment

The cytotoxicity profile of Dtx-MNLC was assessed using sodium 3′-[1-(phenylaminocarbonyl)-3,4-tetrazolium]-bis (4-methoxy-6-nitro) benzene sulfonic acid hydrate (XTT) assay on the MRC-5 and A549 cell lines. For this study, A549 (4 × 103 cells per well) and MRC-5 (5 × 103 cells per well) were seeded in 96-well plates and allowed to grow for 24 h at 37 °C in a humidified incubator with 5% CO_2_. Prior to cellular treatment, Dtx-MNLC was prepared and diluted using the RPMI complete medium with drug concentrations ranging from 10 to 2000 nM (0.008 to 1.616 mg/L). The cell media were carefully discarded without disturbing the cells attached to the plate surface, and a volume of 100 µL of each sample concentration was pipetted into each well in triplicate and incubated for 24 h, 48 h, and 72 h at 37 °C in a humidified incubator with 5% CO_2_. After cell incubation, the sample solution was removed, and each well was washed once with Pbs. Then, 100 μL of cell culture media (CCM) comprised of 90% RPMI, 10% fetal bovine serum, and 1% penicillin-streptomycin was added into each well. Immediately before cell labeling, 4 mL of XTT solution was combined with 10 μL of phenazine methosulfate (PMS) solution. An amount of 25 μL of this mixture was dispensed into each well containing 100 μL CCM for the XTT assay. The cells were then incubated for 2 h at 37 °C and the absorbance was measured afterward using an Agilent BioTek ELISA microplate reader (Agilent, California, CA, USA) at the wavelength of 450 nm. Control wells filled with untreated cells will show the highest absorbance which indicates 100% cell viability. The calculation for the cell viability percentage is shown below.

(4) $$\mathrm{Cell}\;\mathrm{viability}\;\left(\%\right)=\frac{A_{450}\;\mathrm{of}\;\mathrm{sample}\;}{A_{450}\;\mathrm{of}\;\mathrm{control}\;(\mathrm{cell}\;\mathrm{without}\;\mathrm{treatment})}\;\times \;100$$

### 2.7. Statistical Analysis

Data were analyzed in GraphPad Prism version 7 (Dotmatics, Boston, MA, USA) using a two-way ANOVA followed by Tukey’s multiple comparisons to determine the significant difference between the test groups. A statistically significant difference was defined as *p* < 0.05. Data were expressed as the mean ± standard deviation (SD).

## 3. Results and Discussion

### 3.1. Physicochemical Characterisation of Dtx-MNLC

In this work, Dtx-MNLC was prepared using solvent injection technique. The lipids and emulsifiers were selected during component screening prior to the formulation. For the preparation of Dtx-MNLC, solid lipid and oil (Precirol ATO 5 and MCT) with the highest amount of solubilized Dtx were selected and a combination of emulsifiers (TPGS and Lipoid S75) that can produce the smallest nanoparticles were selected. Ethanol and acetone were chosen as the organic phase. Precirol ATO 5 and MCT used in this formulation are generally recognized as safe for human consumption and have been utilized by other studies to formulate lipid nanocarriers. For example, Precirol ATO 5 and MCT were used to prepare NLC to entrap zeaxanthin [25] for lutein delivery [26] and to entrap curcumin for antioxidant delivery [27].

#### 3.1.1. Morphology and Particle Size

**Figure 2:**
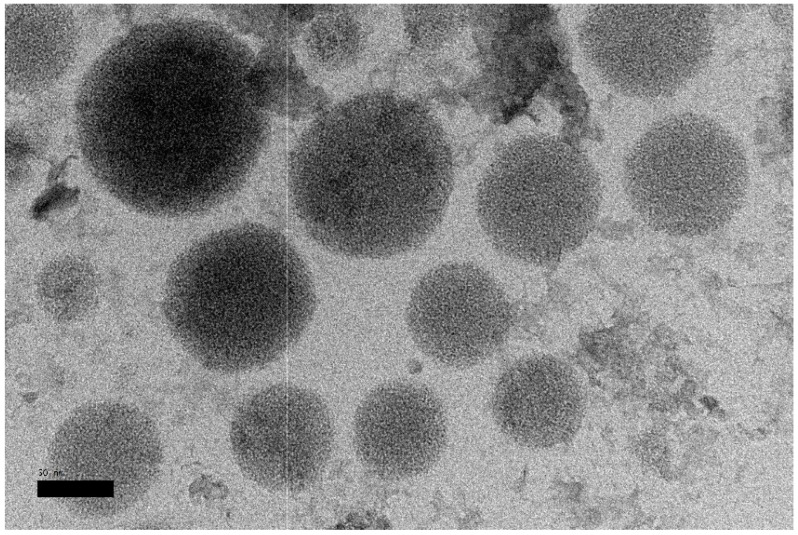
HRTEM image of Dtx-MNLC at 50K× magnification. Scale bar: 50 nm.

The Dtx-MNLC was characterized using HRTEM to observe the shape and size of the nanoparticles. Spherical shapes were observed from the image in Figure 2, but different amounts of IONP entrapped in each particle may contribute to polydispersed particles, as seen by darker contrast on several particles.

The mean size of Dtx-MNLC measured using HRTEM images was 78.5 ± 22 nm, which is smaller than the hydrodynamic diameter measured using the Malvern Zetasizer (110.5 ± 0.1 nm; polydispersity index 0.51 ± 0.02). This size difference may be attributed to the dehydrated state of the nanoparticles due to the sample preparation required for the HRTEM observation. The hydrodynamic diameter of the Dtx-MNLC was measured in its aqueous suspension, whereas the nanoparticles were dried on a carbon-coated copper grid before they were observed under the microscope. Loss of moisture may cause the nanoparticles to shrink to a smaller size. This observation was also reported previously by Kim et al. [28] and Tapeinos et al. [29]. The Dtx-MNLC particle size is within the range of 10 to 200 nm, which is the suggested size for nanocarriers [30]. It is anticipated that therapeutic actives would be delivered more effectively and clearance will be avoided by using nanocarriers in this size range. Apart from mean particle size, the size distribution of the nanocarriers, represented by polydispersity index (PdI), is another crucial parameter for nanoparticle design. The size distribution shows the number of particles in each size segment. A PdI of 0.3 or below is regarded as acceptable in drug delivery applications using lipid-based carriers, and suggests a homogeneous population of phospholipid vesicles, whereas a value above 0.7 suggests a highly broad size distribution [31]. Although size distribution is considered one of the critical quality attributes by the FDA, there is no definition of an acceptable value for PdI in the FDA’s ‘Guidance for Industry’.

#### 3.1.2. Iron Content and Entrapment Efficiency

The amount of IONP and Dtx loaded in the Dtx-MNLC was determined using ICP-OES and HPLC assays, respectively. The developed formulation exhibited high entrapment efficiency of 99.29 ± 1.3%, with a drug loading of 3.98 mg ± 1.2% and a concentration of iron oxide nanoparticles at 0.36 mg/mL. Approximately 0.4% Dtx and 0.36% IONP were entrapped in 1 g of lipid in the Dtx-MNLC formulation. The amount of Dtx loaded in the MNLC was remarkably higher (eight-fold increase) than similar studies for paclitaxel-loaded magnetic solid lipid nanoparticles as reported by Oliveira et al. [32].

#### 3.1.3. Thermal Analysis

**Figure 3:**
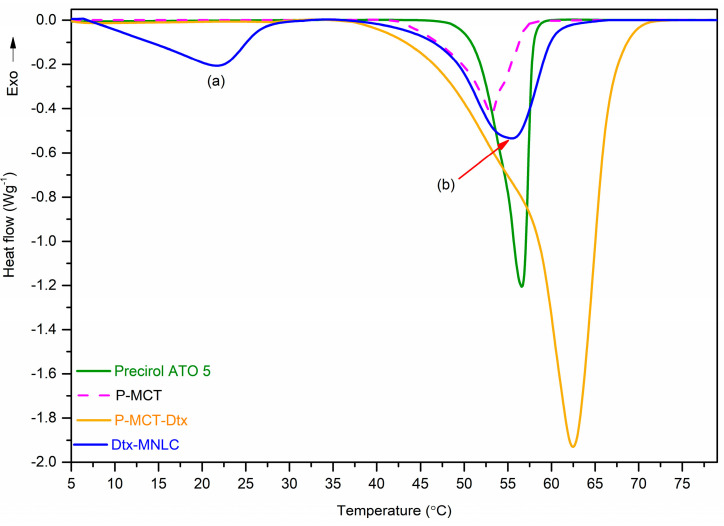
DSC thermogram of Precirol ATO5, binary mixture of Precirol/MCT, ternary mixture of Precirol/MCT/Dtx and formulation of Dtx-MNLC. (a) denotes the first endothermic peak of Dtx-MNLC and (b) is the second endothermic peak.

Figure 3 shows thermograms for the solid lipid (Precirol ATO 5), the binary mixture of Precirol/MCT (P-MCT) in the ratio of 60:40 (*w*/*w*), the ternary mixture of Precirol/MCT/Dtx (P-MCT-Dtx), and the final formulation of Dtx-MNLC measured using DSC. All samples exhibited endothermic peaks, indicating a transition from the solid phase to liquid state occurred at a specific melting point of each sample.

**Figure 4:**
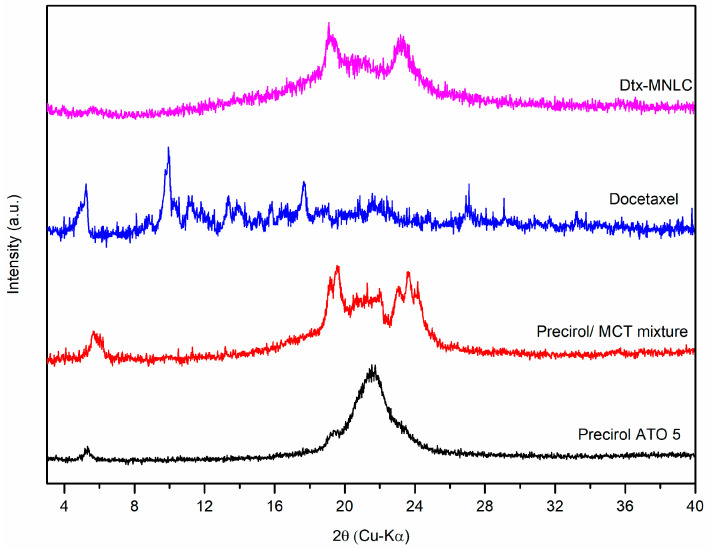
X-ray diffraction pattern of Precirol ATO5, binary mixture Precirol/MCT, Dtx, and Dtx-MNLC.

A sharp endothermic peak exhibited by Precirol ATO 5 at 56.6 °C is consistent with the report by Hamdani et al. [33]. This sharp peak demonstrated the crystalline nature of the solid lipid, which agrees with the PXRD data in Figure 4. The endotherm peak became broader with the addition of MCT in the solid lipid at the ratio of 60:40 *w*/*w* (Precirol/MCT) and the melting point was shifted to a lower temperature (53.1 °C). These changes showed that the MCT has disturbed the crystal lattice arrangement of Precirol, resulting in the lower crystallinity of the solid lipid [34]. Greater disorder in the solid lipid structure when mixed with MCT was also confirmed by the decrease in its melting enthalpy from 97.3 J/g to 96.9 J/g. Incorporation of Dtx (melting temperature: 232 °C) to the mixture of Precirol/MCT increased the melting point to 62.5 °C similar to the observations reported in the published literature [23,35].

Two endothermic peaks were observed in the Dtx-MNLC thermogram, illustrating the presence of a thermodynamically unstable polymorph (indicated by a lower melting temperature) and another more stable polymorph at higher melting point. The first melting event occurred at 22 °C, with a melting enthalpy of 28.2 J/g, while the main peak occurred at 55.5 °C, with a higher melting enthalpy of 65.8 J/g. According to the Ostwald crystallization rule, α-polymorph, being the most unstable crystal with the least amount of molecular conformation, will form first, followed by transformation to a more stable β-form. Complete transformation of α-form to β-form may be partially hindered by the emulsifiers in this work (TPGS and Lipoid S75), acting as ‘polymorphic modifiers’. Some emulsifiers could stabilize the metastable polymorph, thus preventing its complete transformation to a more stable polymorph [36]. Furthermore, the first melting event at 22 °C may be attributed to the melting of Dtx-loaded oil nanocompartments that precipitated near the surface of Dtx-MNLC, possibly located between the emulsifier layers. This may occur especially with Dtx-MNLC prepared with a high content of MCT (as in this work). The MCT may have solubilized and dispersed throughout the solid matrix of Precirol as the lipid cooled down and crystallized, while a smaller part was expelled towards the nanoparticle surface when the oil solubility in the solid lipid matrix exceeded the threshold [37]. Molecular incorporation of MCT in the lipid structure of Precirol can be seen by a broadening of the melting peak at 55.5 °C compared to the sharp peak of pure Precirol as a result of reduced crystallinity due to the distortion of the solid lipid crystal structure [38].

#### 3.1.4. Crystallinity Studies

The X-ray diffraction pattern of Precirol ATO 5, binary mixture of Precirol/MCT, Dtx, and Dtx-MNLC are compared and presented in Figure 4. The PXRD diffractogram of Precirol shows two sharp diffraction peaks at 2θ of 5.3° and 21.4°, indicating a highly crystalline nature of the solid lipid. The disappearance of both peaks in the binary mixture of Precirol/MCT at the ratio of 60:40 (*w*/*w*) and three newly arising peaks at 5.8°, 19.5°, and 23.6° showed that the MCT was distributed homogeneously in the Precirol crystal structure. Peak intensity reduction indicated that there was a change in the crystalline phase of Precirol, demonstrating a disordered and widened lattice structure in the solid lipid crystal when mixed with MCT [39]. This structural defect is advantageous in the development of a nanostructured lipid carrier to improve drug loading and to minimize drug expulsion from the lipid compartment for long-term storage [40].

In the Dtx-MNLC sample, only two characteristic peaks were detected at 19.2° and 23.3°, similar to the peaks of the binary mixture Precirol/MCT. Another peak at 5.8° that was observed in the binary mixture is also seen in the Dtx-MNLC sample, but with a much lower intensity, attributed to a less ordered crystallinity structure in the final formulation of Dtx-MNLC compared to the binary mixture. Higher noise signal in the Dtx-MNLC sample revealed an amorphous fraction compared to the lower noise signal in the crystalline state of Precirol. This amorphous fraction formed by the mixture of emulsifier, MCT, and Dtx inside the solid lipid matrix contributed to the higher drug loading as proposed by Tetyczka et al. [41]. High entrapment efficiency (>99%) as determined previously may be attributed to the complete solubility of Dtx inside the amorphous lipid matrix, evident by the absence of distinctive peaks of Dtx at 5.3°, 13.6°, and 17.7° in the Dtx-MNLC sample.

#### 3.1.5. FTIR Spectroscopy

**Figure 5:**
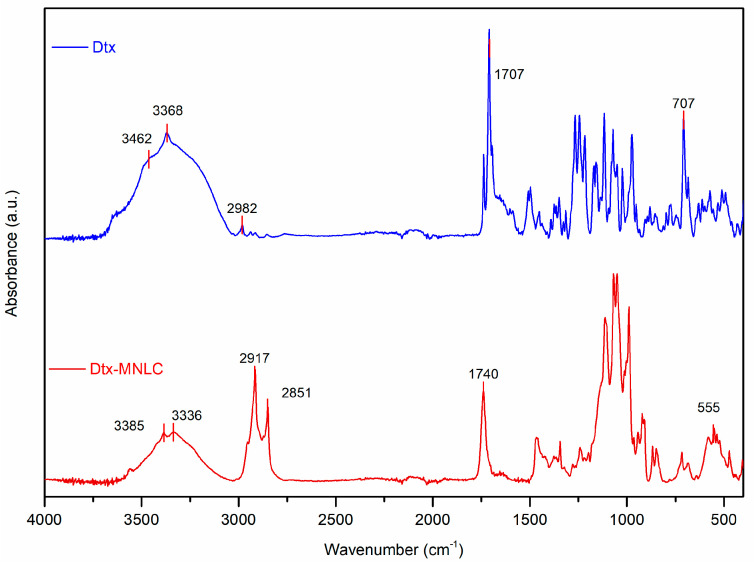
ATR-FTIR spectra of Dtx and Dtx-MNLC.

Infrared spectroscopy (FTIR) was used to verify the entrapment of IONP and Dtx inside the lipid nanoparticles (Figure 5). The stretching vibrations of N-H and O-H in the Dtx sample at 3462 cm^−1^ and 3368 cm^−1^, respectively, were shifted to lower frequencies with lower absorbance intensities (3385 cm^−1^ and 3336 cm^−1^) in Dtx-MNLC samples. The strong band intensity at 1707 cm^−1^ attributed to the presence of a carbonyl group in Dtx was also shifted but to a higher frequency at 1740 cm^−1^ in Dtx-MNLC sample, suggesting an intramolecular hydrogen bond was formed between Dtx and the lipids. A doublet peak at 2917 cm^−1^ and 2851 cm^−1^ indicated the presence of symmetric and asymmetric C-H vibration from the lipid structure of Dtx-MNLC.

Hydrophobic interactions between Dtx and the lipids for successful entrapment of Dtx in the lipid matrix may be observed by the absence of the C-H stretching vibrations at 2982 cm^−1^ and the aromatic C-H out-of-plane bending vibration band at 707 cm^−1^ of Dtx in the Dtx-MNLC sample. A similar pattern of FTIR absorption bands to demonstrate Dtx entrapment in the Dtx-MNLC was reported by Fang et al. [23] in their work to develop an NLC for oral delivery of Dtx.

### 3.2. In Vitro Drug Release

**Figure 6:**
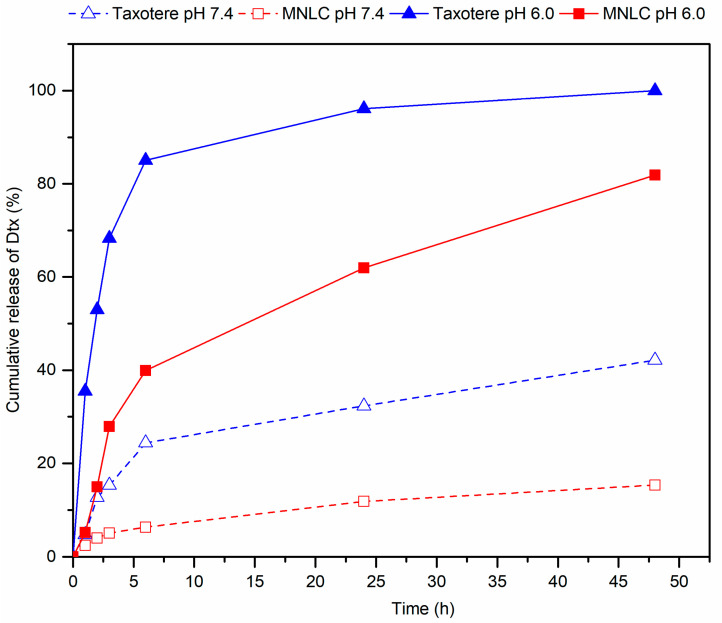
Drug release profiles of CFDtx and Dtx-MNLC using dialysis membrane in SLF at two different pH values. Solid line and dash line represent pH 7.4 and pH 6.5, respectively. The analysis was carried out at 37 ± 1 °C for up to 48 h. Error bars denote standard deviation (*n* = 3). h: hour; CFDtx: commercialized formulation of docetaxel; Dtx-MNLC: docetaxel-loaded magnetic nanostructured lipid carrier.

Drug release profiles of Dtx-MNLC were assessed using a dialysis bag method as described in Section 2.5. The concentration of Dtx diffused through a dialysis membrane into the release media consisting of a fixed volume of simulated lung fluid (SLF) and 0.5% Tween 80 was determined within a 48 h release duration. Pulmonary surfactant in biological lung fluid was not present in the SLF used in this work, thus Tween 80 was added into the release media to increase the solubility of Dtx and to promote diffusion of Dtx through the membrane. The release was conducted in two different pH values; one study was in pH 7.4 to simulate the neutral pH in the body system, the other was carried out in a more acidic environment (pH 6.0) to simulate the condition around cancerous cells. Drug release of Dtx from Dtx-MNLC and a commercialized formulation of Dtx (CFDtx) was studied under the same conditions to compare their release profiles. Both formulations demonstrated a lower release in the simulated extracellular environment of normal tissue (SLF, pH 7.4) than in a simulated intracellular tumor environment in SLF at pH 6.0, as seen in Figure 6. This pattern is in agreement with other publication in the literature, suggesting an accelerated release under acidic condition for lipid-based formulation [42]. Approximately 40% Dtx was released from CFDtx in SLF pH 7.4 within a 48 h period, which is higher compared to Dtx-MNLC (<20% release). In the acidic environment (pH 6.0), Dtx-MNLC shows a biphasic release pattern; rapid release was exhibited during the initial 6 h, with more than 40% release detected in the release media, then a gradual release to 80% was observed until 48 h. On the contrary, the CFDtx shows a cumulative release of more than 90% at the 24 h point.

The difference of the release behavior from CFDtx and Dtx-MNLC can be explained by the formulation composition and its particle structure. CFDtx formed micelles in an aqueous environment due to the presence of Tween 80 surrounding the Dtx core, and rapidly dissociated upon dilution in the release media prior to the release studies [43], thus gradually releasing its cargo through the membrane diffusion. Release profiles characterized by a biphasic behavior were reported from several formulations consisting of solid and liquid lipid cores [23,32,44]. The initial burst release of Dtx may be attributed to the location of Dtx entrapped in the oil nanocompartments near the Dtx-MNLC surface, whereas the more preferential location of Dtx in the deeper lipid core contributed to the subsequent sustained release.

The release data of Dtx-MNLC in SLF pH 6.0 were further fitted into the zero-order (constant release rate), first-order (release amount is dependent on the remaining drugs’ concentration), Higuchi (diffusion-controlled release from solid or semi-solid matrices), Hixson–Crowell, and Korsmeyer–Peppas kinetic models (release from a polymeric matrix) to describe the release mechanism. The assessment of kinetic release was analyzed by applying the linear regression model to obtain the correlation coefficient for each model. The release of Dtx-MNLC was observed to follow the first-order kinetic model, where the plot logarithm of the drug remained in the formulation versus time closely followed the linear fit of the model, having an R^2^ value of 0.9676 as shown in Table 1. The first-order kinetic model was used to describe a concentration-dependent release mechanism, where the release rate depends on the concentration of the remaining drugs in the formulation. The effective therapeutic level can be achieved rapidly from the initial burst release of the drug, then the level is maintained by the well-defined release kinetics (as shown by the release profile in Figure 6). Such release mechanism is advantageous for maintaining the desired drug concentration at the target tissue for a longer time at reduced treatment frequency, which may lead to better patient compliance and a higher rate of successful therapy.

**Figure 7:**
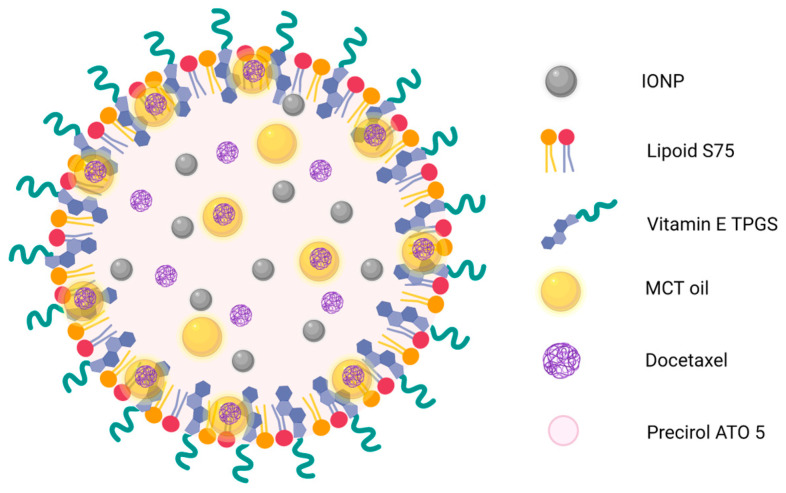
Proposed illustration of Dtx-MNLC structure. IONP: Iron oxide nanoparticles; TPGS: α-Tocopheryl polyethylene glycol succinate; MCT: Medium chain triglyceride.

Based on the results obtained in this work, the following structure is proposed for the Dtx-MNLC, illustrated in Figure 7. IONP may be homogeneously distributed in the solid lipid structure. The theranostic approach via MRI may still be feasible for the formulation, but further studies are needed for confirmation. Dtx was assumed to be entrapped in the solid lipid structure or otherwise solubilized in the MCT, as demonstrated by the drug release profile in Section 3.2. Considering the solubility of MCT in the Precirol may have exceeded the threshold due to the high percentage of MCT in the Dtx-MNLC, some of the oil droplets may be expelled from the lipid crystal and become located near the surface of the Dtx-MNLC, possibly between the surfactant layer of Lipoid S75 and TPGS. The surfactant molecules (Lipoid S75 and TPGS) are packed in a curve structure to form spherical lipid nanoparticles to promote stable dispersion of the particles in the aqueous media.

### 3.3. In Vitro Cytotoxicity Assessment

**Figure 8:**
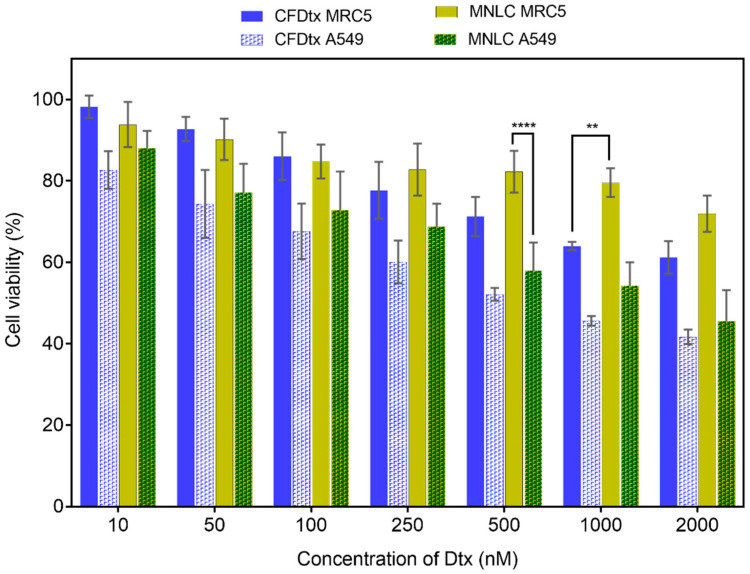
Percentage of cell viability of human lung fibroblast cell lines (MRC5) and human lung carcinoma cell lines (A549) analyzed by XTT assay after treatments with commercialized formulation of Dtx or MNLC formulation at seven different concentrations of Dtx. Cells were incubated post-treatment for 24 h at 37 ± 1 °C. Bars with solid fill and checkered pattern fill represent cell viability of MRC5 and A549, respectively. Error bars denote standard deviation (*n* = 3). Statistical analysis was carried out using a two-way ANOVA test (**** *p* < 0.0001; ** *p* < 0.01). CFDtx: commercialized formulation of docetaxel; MNLC: magnetic nanostructured lipid carrier.

**Figure 9:**
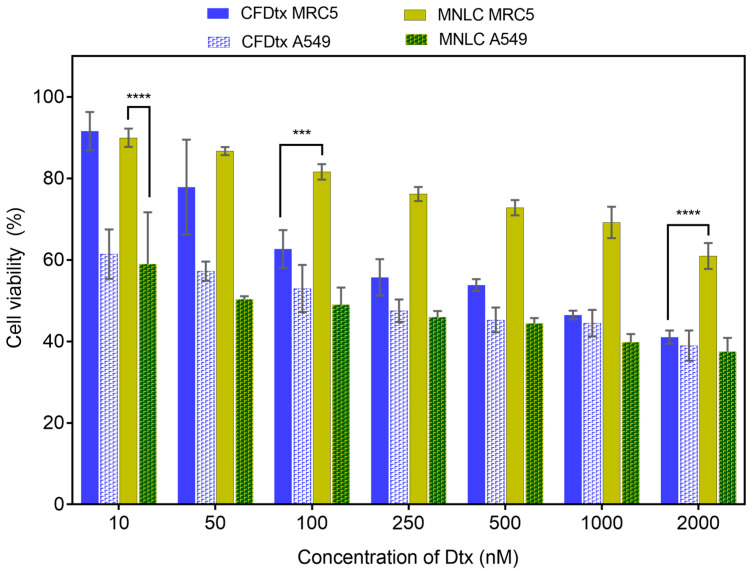
Percentage of cell viability of human lung fibroblast cell lines (MRC5) and human lung carcinoma cell lines (A549) analyzed by XTT assay after treatments with commercialized formulation of Dtx or Dtx-MNLC formulation at seven different concentrations of Dtx. Cells were incubated post-treatment for 48 h at 37 ± 1 °C. Bars with solid fill and checkered pattern fill represent the cell viability of MRC5 and A549, respectively. Error bars denote standard deviation (*n* = 3). Statistical analysis was carried out using a two-way ANOVA test (**** *p* < 0.0001; *** *p* < 0.001). CFDtx: commercialized formulation of docetaxel; MNLC: magnetic nanostructured lipid carrier.

An XTT assay was performed to further investigate the inhibitory effect of the Dtx-MNLC formulation on normal human lung fibroblast cells (MRC5) and human lung adenocarcinoma cells (A549). The effect was quantified by measuring the cell viability after the cells were treated with the formulation and incubated at 24 h and 48 h. Both formulations exhibited a concentration-dependent and time-dependent inhibitory effect as depicted in Figure 8 and Figure 9. According to International Organization for Standardization, a compound is defined to have a cytotoxic effect if the cell growth is inhibited by more than 60% and considered non-toxic if growth inhibition is less than 20%. After 24 h exposure, CFDtx was found to inhibit more cell growth for MRC5 (78% cell viability) than Dtx- MNLC (83% cell viability) at the concentration of 250 nM Dtx, and this inhibitory effect was relatively consistent until the highest concentration tested was reached (2000 nM). However, the percentage of A549 viability was higher at the said concentration when exposed to Dtx-MNLC (69%) compared to CFDtx (60%), although this is not statistically significant. The cytotoxic effect of Dtx-MNLC on A549 was significant (*p* < 0.001) compared to MRC5 above the concentration of 500 nM, indicating higher intracellular Dtx concentration in the adenocarcinoma cells than normal cells. These results correlate with the drug release study previously discussed in Section 3.2, that about 60% Dtx was released from Dtx-MNLC upon 24 h exposure to an acidic environment such as around cancerous cells than in a normal cell environment (approximately 15%).

Cell exposure to the formulations was prolonged until 48 h to further observe the cytotoxicity effects. According to the drug release studies, CFDtx reached a 100% cumulative release at 48 h, whereas about 82% release was detected from Dtx-MNLC at the same timepoint. Nevertheless, it is interesting to note that ANOVA analysis shows the overall difference of A549 cell deaths treated with either of the formulations was not statistically significant, but a reduced toxicity towards MRC5 by Dtx-MNLC was significant (*p* < 0.001) compared to CFDtx, especially at the concentrations above 100 nM. Dtx-MNLC induced a significant cytotoxicity effect on A549 by inhibiting more than 40% cell growth even at the lowest concentration (10 nM), but not to MRC5 (*p* < 0.0001).

For further comparison, the concentration of Dtx in each formulation required to inhibit 50% of cell growth (IC_50_) was calculated for each cell line and each incubation time, as listed in Table 2. A higher value of IC_50_ for Dtx-MNLC than for CFDtx after 24 h of exposure to A549 correlates with a lower percentage of drug release of Dtx-MNLC in Section 3.2. During the drug release study, approximately 60% of Dtx was released from Dtx-MNLC, compared to a >90% release from CFDtx. Consequently, a higher dose of Dtx was required in the Dtx-MNLC formulation than CFDtx to inhibit 50% of A549 cell growth. However, Dtx-MNLC demonstrates better safety towards MRC5 at both 24 and 48 h exposure, with increased cytotoxicity towards A549 compared to CFDtx. The results indicate that a much lower concentration of Dtx in Dtx-MNLC may be required to cause the same number of A549 cell deaths in comparison with the commercial formulation of Dtx.

The cytotoxic effect of a drug formulation depends on the composition and intracellular drug concentration. Cellular internalization of nanoparticles in vitro and in vivo occurs through various uptake routes, categorized into (i) endocytosis-based uptake and (ii) direct cellular entry [45]. Cells usually take up nanoparticles through endocytosis and confine them within the endocytic vesicles, preventing them from directly accessing the cytoplasm. Lipid nanoparticles release their therapeutic payload via endosomal escape, likely due to the ionizable properties of the lipid at a low pH [46]. Non-specific cell membrane permeability in drug formulations containing Tween 80 as CFDtx may potentially induce a cytotoxic effect on both normal cells and cancer cells [47] as demonstrated in this work. The use of TPGS in the formulation has been reported to enhance cellular uptake by effectively inhibiting P-glycoprotein (P-gp) activity in A549, thus increasing the cytotoxic effect of Dtx-MNLC on the cancerous cells [48,49]. P-gp is located on the surface of the epithelial membrane and is responsible for preventing the intracellular accumulation of foreign substances, including drugs. This P-gp membrane transporter is overly expressed in cancerous cells, leading to reduced bioavailability and efficacy of chemotherapeutics in the tumor cells, and is often linked to multidrug resistance at the cellular level. Inhibition of the P-gp efflux pump by drugs and surfactants such as TPGS has been reported to increase cellular retention of anticancer agents, enhancing the therapeutic efficacy of the chemotherapy [50,51]. Furthermore, higher cytotoxicity of Dtx-MNLC on A549 may be attributed to selective apoptogenic activity in tumor cells documented for TPGS. Upon uptake in the tumor cells, it can target the mitochondria, causing mitochondrial destabilization and activating the mitochondrial mediator for cell apoptosis [49]. Interestingly, this mechanism was not reported to occur in normal cells, which explains its lower toxicity towards healthy tissues. The synergistic effect of TPGS, Dtx, and IONP in Dtx-MNLC may hypothetically increase the therapeutic activity on A549 cells and reduce toxicity towards MRC5 compared to the commercial formulation of Dtx.

## 4. Conclusions

This study demonstrates that the IONP and Dtx were successfully entrapped in the magnetic nanostructured lipid carrier. Physicochemical characterization of the Dtx-MNLC showed that the particles were spherical with a mean particle size of approximately 100 nm after purification. Combination of MCT and Precirol ATO 5 has disturbed the crystalline arrangement of pure Precirol ATO 5, resulting in successful Dtx loading and IONP in the lipid structure. In vitro drug release studies in SLF media demonstrate higher release in a simulated cancerous environment (acidic condition) than in the neutral pH of the healthy tissues. In the SLF with a pH of 6.0, initial burst release during the first 6 h was observed, followed by sustained release up to 48 h. The kinetic release was found to fit the first-order kinetic model. Safety and efficacy studies of Dtx-MNLC were conducted on A549 and MRC5 using an XTT assay and compared with the commercial formulation of Dtx. Both formulations exhibited dose-dependent and time-dependent cytotoxicity during 24 and 48 h exposure. Dtx-MNLC demonstrates higher cytotoxicity on A549 (IC_50_ = 855 nM) than CFDtx (IC_50_ = 551 nM), while showing a less cytotoxic effect on MRC5. However, the cytotoxicity studies were limited to an in vitro assay, and the safety and efficacy of this formulation has not yet been demonstrated in vivo. Further studies should also include investigation into the polymorphic stability of this formulation over a defined storage time to ensure a drug’s efficacy can be retained within a longer period of storage. This study demonstrates that the developed magnetic nanostructured lipid carrier loaded with Dtx and IONP has the potential to be used as diagnostic and therapeutic agent in lung cancer treatment. Monitoring of therapeutic efficacy in the tumor may be accomplished using MRI, and the reduced toxicity of the formulation on MRC5 showed a promising alternative to the commercialized formulation of Dtx.

## Figures and Tables

**Table 1:** Model fitting for release kinetics of Dtx-MNLC.

Formulation	Correlation Coefficient of Model (*R*^2^)				
	Zero-Order	First-Order	Higuchi	Hixson–Crowell	Korsmeyer–Peppas
Dtx-MNLC	0.8495	0.9676	0.9625	0.9383	0.9436

**Table 2:** IC_50_ value of MRC5 and A549 cell lines after 24 and 48 h exposure to CFDtx and Dtx-MNLC.

Sample	IC_50_ (nM)			
	24 h		48 h	
	MRC5	A549	MRC5	A549
CFDtx	1877	551	527	251
Dtx-MNLC	3718	855	1999	178

IC_50_: the half-maximal inhibitory concentration; Dtx: docetaxel; A549: human lung carcinoma; MRC5: human lung fibroblast cells.

## Data Availability

All data relevant to the publication are included.

## Abbreviations

A549	Human lung adenocarcinoma cells
ATR-FTIR	Attenuated total reflectance- Fourier transform infrared
CFDtx	Commercial formulation of docetaxel
CT scan	Computed tomography scan
DDS	Drug delivery system
DSC	Differential Scanning Calorimetry
Dtx	Docetaxel
Dtx-MNLC	Docetaxel-loaded magnetic nanostructured lipid carrier
HCl	Hydrochloric acid
HR-TEM	High-resolution transmission electron microscope
ICP-OES	Inductively coupled plasma-optical emission spectrometry
IONP	Iron oxide nanoparticle
MCT	Medium chain triglyceride
MRC-5	Human lung fibroblast cells
MRI	Magnetic resonance imaging
NLC	Nanostructured lipid carrier
OA-IONP	Oleic acid-coated iron oxide nanoparticle
ODE	1-octadecene
PdI	Polydispersity index
P-gp	P-glycoprotein
P-MCT	Mixture of precirol and medium chain triglyceride
P-MCT-Dtx	Ternary mixture of precirol, medium chain triglyceride and docetaxel
PXRD	Powder X-ray diffraction
siRNA	Small inhibitory RNA
SLF	Simulated lung fluid
SLN	Solid lipid nanoparticle
TPGS	D-a-tocopheryl polyethylene glycol succinate
